# Decision-making Among Hepatitis C Virus-negative Transplant Candidates Offered Organs from Donors with HCV Infection

**DOI:** 10.1097/TXD.0000000000001341

**Published:** 2022-07-19

**Authors:** M. Elle Saine, Erin M. Schnellinger, Michel Liu, Joshua M. Diamond, Maria M. Crespo, Stacey Prenner, Vishnu Potluri, Christian Bermudez, Heather Mentch, Michaella Moore, Behdad Besharatian, David S. Goldberg, Frances K. Barg, Peter P. Reese

**Affiliations:** 1 Department of Biostatistics, Epidemiology, and Informatics, Perelman School of Medicine, University of Pennsylvania, Philadelphia, PA.; 2 Leonard Davis Institute of Health Economics, University of Pennsylvania, Philadelphia, PA.; 3 Renal-Electrolyte and Hypertension Division, Perelman School of Medicine, University of Pennsylvania, Philadelphia, PA.; 4 Pulmonary, Allergy, and Critical Care Division, Perelman School of Medicine, University of Pennsylvania, Philadelphia, PA.; 5 Gastroenterology Division, Perelman School of Medicine, University of Pennsylvania, Philadelphia, PA.; 6 Department of Surgery, Perelman School of Medicine, University of Pennsylvania, Philadelphia, PA.; 7 Department of Biology, College of Arts and Sciences, Howard University, Washington, DC.; 8 Department of Medicine, Division of Hepatology, University of Miami Miller School of Medicine, Miami, FL.; 9 Department of Family Medicine and Community Health, University of Pennsylvania Perelman School of Medicine, Philadelphia, PA.

## Abstract

**Background.:**

Historically, many organs from deceased donors with hepatitis C virus (HCV) were discarded. The advent of highly curative direct-acting antiviral (DAA) therapies motivated transplant centers to conduct trials of transplanting HCV-viremic organs (nucleic acid amplification test positive) into HCV-negative recipients, followed by DAA treatment. However, the factors that influence candidates’ decisions regarding acceptance of transplant with HCV-viremic organs are not well understood.

**Methods.:**

To explore patient-level perceptions, influences, and experiences that inform candidate decision-making regarding transplant with organs from HCV-viremic donors, we conducted a qualitative semistructured interview study embedded within 3 clinical trials investigating the safety and efficacy of transplanting lungs and kidneys from HCV-viremic donors into HCV-negative recipients. The study was conducted from June 2019 to March 2021.

**Results.:**

Among 44 HCV-negative patients listed for organ transplant who were approached for enrollment in the applicable clinical trial, 3 approaches to decision-making emerged: positivist, risk analyses, and instinctual response. Perceptions of risk contributed to conceptualizations of factors influencing decisions. Moreover, most participants relied on multiple decision-making approaches, either simultaneously or sequentially.

**Conclusions.:**

Understanding how different decisional models influence patients’ choices regarding transplant with organs from HCV-viremic donors may promote shared decision-making among transplant patients and providers.

Transplant candidates vastly outnumber donated organs.^1^ Historically, transplant teams discarded organs from donors infected with hepatitis C virus (HCV) because of the risk of graft failure and poor patient outcomes associated with chronic infection.^2,3^ Highly effective, all-oral direct-acting antiviral (DAA) therapies revolutionized HCV management because of the more tolerable side-effect profiles and cure rates >95%.^4,5^ These therapies support new possibilities for increasing donor organ availability by transplanting HCV-viremic organs into HCV-negative recipients.^5^

Concurrently, the opioid epidemic has increased the number of organs donated by HCV-viremic deceased donors.^6-9^ In 2016, clinical trials began investigating the safety and efficacy of transplanting HCV-viremic organs (nucleic acid amplification test positive) into HCV-negative recipients.^10,11^ Early trials and single-center case series reported cure rates of 96%–100%,^10-18^ and among kidney transplant recipients, 12-month allograft function was comparable with transplants from HCV-negative donors.^9,12,19^

Waitlisted patients are increasingly willing to accept transplants from HCV-viremic donors.^1,20^ Consequently, investigators have called for patient-centered research promoting rigorous informed consent and patient education,^5,21-24^ including evaluation of transplant candidates’ informational needs and decision-making processes surrounding HCV-viremic donor organs.^19,21,25^ To date, only one study^26^ assessed the experiences of HCV-negative recipients transplanted with HCV-viremic organs. Among 8 kidney transplant recipients, risk/benefit assessments were found to inform decisions,^26^ yet how decision-making differs between trial decliners and enrollees remains unknown. Additionally, although there is a rich body of literature surrounding patient decision-making in clinical trial enrollment,^27-30^ much of the most in-depth research on clinical trial participation comes from the cancer literature. We seek to expand understandings of decision-making by focusing on the distinct experiences of patients waitlisted for organ transplant. Moreover, decision-making regarding HCV-viremic organs poses unique challenges beyond traditional “high-risk organs” because considerable disease stigma surrounds HCV,^31^ approaches to educating patients about the risks and benefits of HCV-viremic organs are not standardized^32,33^ and may be incomplete,^34^ and access to DAAs may not be guaranteed to all patients in routine clinical practice due to variations in insurance coverage.^32,35-37^

Qualitative research is ideal for evaluating experiences and factors that matter most to patients and when undertaken in the context of clinical trials, can improve understanding of how participants perceive the trial and facilitate the interpretation of results.^38^ This is especially true in the field of organ transplantation.^39^ To identify perceptions and decision-making processes among transplant candidates who were approached to receive organs from HCV-viremic donors, we conducted a qualitative study embedded within 3 clinical trials. We interviewed patients at all stages of transplantation, including those who declined HCV-viremic donor organs. Both kidney and lung transplant patients were included to investigate how organ type influences decision-making and informed consent (eg, due to differences in illness acuity illness or organ allocation).

## MATERIALS AND METHODS

### Study Design and Setting

From June 2019 to March 2021, we conducted an exploratory qualitative study embedded within 3 open-label clinical trials investigating the safety and efficacy of transplant from HCV-viremic donors into HCV-negative patients, followed by DAA therapy. Two trials were conducted among kidney transplant candidates (Multicenter Study to Transplant Hepatitis-C Infected Kidneys [MYTHIC; ClinicalTrials.gov: NCT03781726] and Transplanting Hepatitis C Kidneys Into Negative Kidney Recipients [THINKER; NCT02743897]). The third trial was conducted among lung transplant candidates (Open-Labeled Trial of Zepatier for Treatment of Hepatitis C-Negative Patients who Receive Lung Transplants from Hepatitis C-Positive Donors [SHELTER; NCT03724149]). SHELTER and THINKER are single-center studies; MYTHIC was conducted across 7 transplant centers. Trial screening criteria varied due to protocol differences (see **SDC I** http://links.lww.com/TXD/A436); across all 3 trials, eligible participants were adult patients listed for organ transplant who were expected to tolerate DAA therapy, could provide informed consent, and had no evidence of active liver disease. Clinical trial enrollment required (1) initial contact with an investigator; (2) formal presentation of the risks/benefits of transplantation, including information about HCV; and (3) in-person consent and evaluation. Our qualitative study was approved by the Institutional Review Board (Protocol #833364).

### Participants and Recruitment

Our embedded qualitative study was conducted between June 2019 and March 2021 within 3 ongoing, open-label clinical trials (SHELTER, MYTHIC, and THINKER) investigating the safety and efficacy of transplant from HCV-viremic donors into HCV-negative patients, followed by DAA therapy. All participants who were actively enrolled in the SHELTER, MYTHIC, and THINKER trials at the University of Pennsylvania before June 2019 were invited to participate in our qualitative interview study. Moving forward, all participants who were subsequently approached for formal education and enrollment in the SHELTER, MYTHIC, and THINKER trials—including those who were approached by an investigator and declined to enroll—were eligible for inclusion in our qualitative study. Assenting patients were contacted by phone and provided consent verbally (the Institutional Review Board waived the need for written informed consent). Patient characteristics were extracted from their electronic medical record and entered into REDCap for analyses. Interviews lasted approximately 15–30 min. Following the interview, participants received a $20 gift card via mail.

**FIGURE 1.**
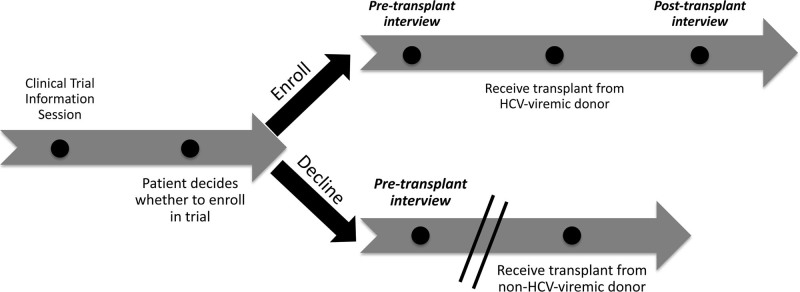
Timeline depicting relationship between participants enrolled in the embedded qualitative study to the associated clinical trials. Interviews were conducted at 2 potential timepoints: pretransplant or posttransplant. Pretransplant interviews were conducted among both trial enrollees and trial decliners. Bold, italic text indicates potential timepoints for interviews. Break lines indicate the end of follow-up for the qualitative interview study. Posttransplant interviews were only conducted among participants who received a transplant from an HCV-viremic donor. HCV, hepatitis C virus.

Figure 1 displays trial enrollment steps and the conduct of qualitative interviews. The clinical trial information session contained educational slides about HCV infection and treatment provided through the parent clinical trials (see **SDC II** http://links.lww.com/TXD/A436 for more details). We sampled patients purposively to maximize variation by organ type (kidney and lung) and transplant stage (pretransplant, posttransplant, and declined). Recruitment continued until thematic saturation (see the “Analyses” section for more details).^40-42^ The pretransplant interview questions were asked of all participants, regardless of whether they enrolled or declined the associated clinical trial. The posttransplant interview was only conducted among participants who received a transplant from HCV-viremic donors through one of the associated clinical trials (Figure 1). Most participants were interviewed within 1 y of their initial contact date or trial enrollment date (Table 1; **Table S2, SDC** http://links.lww.com/TXD/A436).

**TABLE 1.** Characteristics of participants, overall, and by trial participation status

Characteristics	Overall (n = 44)	Pretransplant (n = 14)	Posttransplant (n = 18)	Declined (n = 12)
Clinical trial (n, %)				
MYTHIC/THINKER (kidney transplant)	33 (75%)	10 (71%)	12 (67%)	11 (92%)
SHELTER (lung transplant)	11 (25%)	4 (29%)	6 (33%)	1 (8%)
Time elapsed (median [IQR])				
Days between index date^a^ and interview	283.5 (63.5, 363.5)	184 (47, 539)	337 (268, 378)	63.5 (39.5, 318)
Days between clinical trial enrollment date^b^ and interview	—	178 (35, 539)	328.5 (243, 363)	—
Age (y) at time of interview (median [IQR])	55.0 (49.5, 60.5)	52.0 (41.0, 59.0)	57.0 (53.0, 61.0)	55.5 (53.5, 59.5)
Sex (n, %)				
Male	31 (70%)	9 (64%)	10 (56%)	12 (100%)
Female	13 (30%)	5 (36%)	8 (44%)	0 (0%)
Race (n, %)				
Asian	2 (5%)	0 (0%)	1 (6%)	1 (8%)
Black/African American	12 (27%)	2 (14%)	5 (28%)	5 (42%)
White	27 (61%)	10 (71%)	11 (61%)	6 (50%)
Other/missing value	3 (7%)	2 (14%)	1 (6%)	0 (0%)
Ethnicity (n, %)				
Not Hispanic/Latino	42 (95%)	12 (86%)	18 (100%)	12 (100%)
Hispanic/Latino	2 (5%)	2 (14%)	0 (0%)	0 (0%)
Education (n, %)				
Less than high school degree	2 (5%)	0 (0%)	2 (11%)	0 (0%)
High school degree/GED	6 (14%)	1 (7%)	1 (6%)	4 (33%)
Associated degree/some college	12 (27%)	3 (21%)	6 (33%)	3 (25%)
College/graduate degree	17 (39%)	9 (64%)	5 (28%)	3 (25%)
Other/missing value	7 (16%)	1 (7%)	4 (22%)	2 (17%)
Employment (n, %)				
Unemployed or disability	22 (50%)	6 (43%)	10 (56%)	6 (50%)
Employed	13 (30%)	4 (29%)	5 (28%)	4 (33%)
Retired	6 (14%)	3 (21%)	1 (6%)	2 (17%)
Other/missing value	20 (45%)	1 (7%)	2 (11%)	0 (0%)
Living situation (n, %)				
Alone	3 (7%)	2 (14%)	1 (6%)	0 (0%)
Lives with spouse or significant other	28 (64%)	7 (50%)	11 (61%)	10 (84%)
Lives with other family	8 (18%)	3 (21%)	4 (22%)	1 (8%)
Other/missing value	5 (11%)	2 (14%)	2 (11%)	1 (8%)

^a^Index Date refers to the date each participant was first contacted by the clinical trial team, and is applicable to all study participants.

^b^Enrollment Date refers to the date each participant completed clinical trial screening or the education session (whichever occurred first), and is only applicable to participants enrolled in the clinical trial.

GED, general educational development; MYTHIC, Multicenter Study to Transplant Hepatitis-C Infected Kidneys; SHELTER, Open-Labeled Trial of Zepatier for Treatment of Hepatitis C-Negative Patients who Receive Lung Transplants from Hepatitis C-Positive Donors; THINKER, Transplanting Hepatitis C Kidneys Into Negative Kidney Recipients.

### Data Collection

Semistructured telephone interviews explored (1) differences in attitudes and beliefs between those who accepted organs from HCV-viremic donors and those who declined and (2) patient experiences with transplant. Our approach builds upon the Integrated Behavior Model (IBM),^43,44^ which examines how attitudes, perceptions of social norms, and personal agency influences intention to adopt a behavior, given sufficient knowledge and resources. **SDC III,** http://links.lww.com/TXD/A436 describes how we adapted the IBM model to our clinical context. All interviewees were asked the same core set of open-ended questions (**SDC IV** http://links.lww.com/TXD/A436). Posttransplant interviewees were asked about their posttransplant health; kidney patients were asked about dialysis experiences.

M.E.S., E.M.S., M.L., and M.M. conducted semistructured telephone interviews. All interviewers are women trained in qualitative data collection and analyses, hold bachelor’s degrees or higher, and are uninvolved in the clinical trials. F.K.B. (female anthropologist with mixed-methodology expertise) and P.P.R. (male kidney transplant physician, clinical trial principal investigator with epidemiology expertise) supervised the team (see **SDC V** http://links.lww.com/TXD/A436 for Consolidated Criteria for Reporting Qualitative Studies [COREQ] checklist).^45^

### Analyses

Interviews were audio-recorded and transcribed (M.L. and M.M.), de-identified, and uploaded to NVivo 11 (QSR International 2013, Doncaster, Australia). Transcripts underwent multiple rounds of deductive and inductive coding using a modified grounded theory approach.^46,47^ A priori codes based on the IBM were applied to all data, followed by independent (M.E.S. and E.M.S.) line-by-line reading of transcripts to identify emerging themes. Relationships among inductive codes (eg, meta-themes and thematic variation) were described using axial codes. Coding discrepancies were resolved via discussion among the authors. Thematic saturation was evaluated via the Framework Method, which involves summarizing emerging themes and “charting” data in a spreadsheet to visualize patterns within and across respondents.^42^ Trustworthiness was also enhanced by peer debriefing.

Patient characteristics were extracted from the electronic medical record and summarized using counts (proportions) for categorical variables and medians (interquartile ranges) for continuous variables (STATA 15, StataCorp LLC, College Station, TX).

## RESULTS

We attempted to contact 61 patients, of whom 44 participated, 3 declined, and 14 were unreachable after at least 5 contact attempts. Thematic saturation for the overall sample was reached after 15 interviews. We interviewed 44 participants (11 [25%] lung, 33 [75%] kidney) to achieve saturation across subgroups (declined, pretransplant, and posttransplant) and maximize representativeness of the clinical trial subpopulation. Most participants were male (70%) and white (61%), with a median age of 55 years. At the time of the interview, 14 respondents (32%) were pretransplant, 18 (41%) were posttransplant, and 12 (27%) declined participation in the clinical trials (Table 1). Index date defined the date that participants were first contacted by the clinical trial team. The mean number of days between the index and interview date was longer among patients interviewed posttransplant (337 d) compared with those interviewed after declining participation in the clinical trials (63 d) as posttransplant respondents were only interviewed after they received transplant and recovered from transplant surgery (ie, wait-time and recovery time are included in the mean number of days between index and interview date for posttransplant interviewees but not for those who declined participation in the clinical trials). **Table S1, SDC,** http://links.lww.com/TXD/A436 provides additional characteristics by the organ transplant type.

### Conceptual Model

**FIGURE 2.**
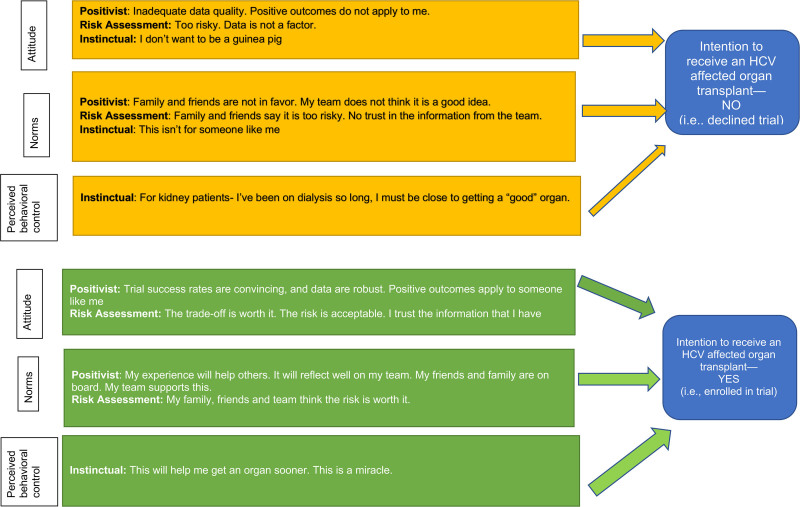
Conceptual model that maps the decision-making approaches that emerged from our interviews to the IBM. IBM, integrated behavior model. HCV, hepatitis C virus.

Three decision-making approaches emerged: positivist, risk assessment, and instinctual response. These themes were mapped to the “attitudes,” “norms,” and “perceived behavioral control” domains of the IBM (Figure 2). We stratified this conceptual model by patients’ choice to enroll in or decline the trial, which is equivalent to stratifying by patients’ intention to receive organ offers from HCV-viremic donors or not, respectively. Contextual factors influencing decisions included attitudes toward research, input from others, waitlist time, and perceptions of organ quality.

Multiple decision-making approaches were associated with each IBM domain, suggesting that decision-making approaches can complement each other and are not mutually exclusive. Kidney and lung transplant candidates endorsed similar decision-making approaches regardless of when they were interviewed (pretransplant, posttransplant, and declined) or whether they ultimately chose to enroll in or decline the trial, lending further credibility to the reliability of our data and the robustness of our conceptual model. **SDC III** http://links.lww.com/TXD/A436 provides additional details on how we used the IBM to develop our interview guide and construct our conceptual model. We discuss each decision-making approach below and support each with exemplar quotes from interview respondents to illustrate the diversity of perspectives on patient decision-making surrounding HCV-viremic donor organs and the nuances behind such decisions. To show how the same decision-making approach may lead to a different decision outcome between enrollers and decliners, we discuss each decision-making approach separately by clinical trial enrollment status. It is important to emphasize that these approaches are not mutually exclusive and participants may move between approaches during the process of making a decision.

### Decision-making Approaches Among Patients who Enrolled in the Trial

#### Positivist Approaches

Many patients adopted positivist approaches that evaluated potential clinical outcomes, often employing probabilities and outcome data (eg, trial success rates), in their decision-making. By “positivist,” we mean a decision-making style that relies on scientific evidence. For example, some respondents referenced outcome data as evidence that the trials were successful: *“*I think 90 some people had it done… it had been done before so I knew it could be a success…I just figured this is the only chance I’m gonna get” [kidney; posttransplant]. For many, important outcomes included both successful transplant and treatment of HCV without complication. Specifically, trial enrollees were confident that DAA therapies could “eliminate” HCV from their bodies, citing a “99%” likelihood of cure: “for me, [it] is no different than getting a kidney from a person without hepatitis C because it’s as simple as treating me with a medication that the science is already treating with and having a 99% rate of success” [kidney; pretransplant].

Although some patients did their own research, most relied on their medical team for information to guide their decision-making. Patients who endorsed less prior knowledge about HCV described how the education session aided their decision by increasing their knowledge, answering questions, and providing data: “Education is powering” [lung; posttransplant]. Notably, not all patients found the education session helpful. For example, 2 posttransplant lung patients felt the amount of information conveyed in the session was overwhelming, highlighting how, for some patients, ongoing discussions may be more important to informed consent than a single education session.

#### Risk Analyses

Some patients relied on weighing potential risks and benefits in their decision-making. For example, kidney transplant participants frequently contextualized their risk/benefit assessment within their dialysis experience. The potential for receiving an organ sooner and/or from younger donors was appealing to patients who viewed the trial as a means to liberate themselves from dialysis, avoid transitioning from peritoneal to hemodialysis, or circumvent dialysis altogether: “If I waited without getting into this program, I would get worse-graded kidney and wouldn’t be able to wait without going into dialysis, which would affect it to an even greater degree” [kidney; posttransplant]. Others viewed potential health risks associated with HCV-viremia as less concerning than those arising from long-term dialysis: “I was afraid [that] what dialysis was doing to my body could potentially be worse than getting a contaminated kidney” [kidney; posttransplant].

Some patients framed transplant as an inherent trade-off of risks, with the goal of an ultimate net-positive result. For example, one enrollee explained how HCV-viremia was being traded for their end-organ disease: “when it comes to transplant, you’re basically trading one set problems for another” [lung; pretransplant]. For others, the benefits to society through contribution to research outweighed personal risk: “hopefully they learn something from me, I’m part of a study, a dataset; I’m a datapoint. It may prove something positive for someone else. So I agreed to do it” [kidney; posttransplant]. Compared with kidney transplant recipients, lung transplant recipients more frequently described the potential for societal good to come from their participation: “I don’t normally turn down any kind of program because if it’s for the benefit – not necessarily me, but someone later in life, then that’s great. I’m more out to kind of help the future than myself right now. But if it helps me at the same time, that’s awesome” [lung; posttransplant]. Several patients delineated their threshold for acceptable risk as it pertains to contracting HCV and curability: “they said 99% of the people participate get cured of hep C...I’m a gambling man, I love blackjack, poker, you know, betting on sports, so I’ll take 99%. Anything over 90 is pretty much 100 to me, even though I know there’s still a risk. But as a gambler, if I hear over 90, you might as well tell me 100. I’m gonna go for it” [kidney; posttransplant]. However, patients varied in their threshold for acceptable risk.

Engagement with the trial team also influenced patients’ decisions; patients valued transplant teams who seemed fully transparent about the risks of accepting HCV-viremic donor organs. Feelings of safety were linked to patients’ trust in science and medicine: “I don’t believe that somebody is going to transplant a kidney into me that wouldn’t be good for me. I trust the doctors, I trust science, I trust the hospital, and with that trust, I’m believing that I’m going to be safe with that transplant” [kidney; pretransplant]. Providing information in accessible language—not medical jargon—facilitated patients’ understanding of the risks, benefits, and consequences of accepting such organs. Patients’ decisions to participate in the trial were strongly associated with whether they felt that the trial team addressed their questions and concerns about transplant with HCV-viremic organs. Patients who trusted the transplant team to provide good pretransplant and posttransplant care had more confidence and were more comfortable accepting HCV-viremic donor organs. Reciprocally, some patients with strong trust in their teams expressed a desire to “do well with transplant” [kidney; pretransplant] for their transplant team and based their decision to participate in the trial on whether they thought doing so would allow them to contribute to the transplant team’s success.

Input from family and friends, including other patients (eg, at dialysis), was also central to many patients’ decisions. Most patients described participating in a form of shared decision-making with loved ones. However, for some enrollees, input from loved ones conflicted with their own decision to undergo transplant; consequently, some patients avoided discussions with others to minimize negative input, whereas others emphasized that the decision was ultimately their own: “ultimately, it’s not anybody else’s decision… as much as you want other people to make the decision for you, they don’t and they won’t. You have to kind of weigh all your options and make the decision yourself” [kidney; pretransplant]. For others, knowing others who had successfully been treated for HCV helped mitigate concerns regarding HCV-associated health risks: “I was wary at first, but then after talking to my family, I never realized that my one brother had hepatitis C, and that he had gotten the medication. He was cured from it. So knowing that, and just looking into it, made me feel as though my chances are better if I sign up for the research” [lung; posttransplant].

#### Instinctual Approaches

Not all participants relied on cognitive models to work through their choices and reach a decision. Multiple patients described a gut-response or instinctual reaction to the clinical trial invitation, saying they made their decision almost instantaneously: “we have two absolutely miracle drugs that totally eliminate hepatitis C... it took me about one tenth of one second to say yes” [kidney; posttransplant]. Notably, patients who made their choice instinctually often utilized cognitive decision processes to support their initial reaction, consistent with the notion that decision-making approaches are not mutually exclusive: “I think it [the education session] solidified the way I was already leaning and then hearing that the outcomes were doing so well that it just solidified that” [lung; pretransplant]. For these patients, additional information may help rationalize their choice but may be less likely to form or change their decision.

### Decision-making Approaches Among Patients who Declined the Trial

#### Positivist Approaches

Patients who relied on positivist approaches and declined trial participation described concerns of small sample sizes and inadequate follow-up: “I know you’ve only done three lung transplants that took the virus. So, if you had a bigger sample size for me…But my strength are in numbers” [lung; declined]. Likewise, decliners expressed reservations regarding the safety and efficacy of DAA therapies in their own bodies: “Although they can treat that [hepatitis C] with high success rates, I don’t think the research shows enough long-term effects of those medications. Nor do I know if I’ll be able to tolerate those particular medications that are used to treat hep C in the likely event I’ll get hep C. So that is what has caused me reservations” [kidney]. Moreover, decliners who attended the education session more often expressed lingering questions, including unanswerable unknowns.

#### Risk Analyses

Dialysis experience played a lesser role in the risk perceptions of decliners, although some patients who had accumulated considerable time on dialysis felt that they must be “closer” to receiving a non-HCV-viremic donor organ, and therefore were not willing to accept the potential risks: “That’s given me hope that I’m getting close and I don’t need to resort to taking a higher risk kidney” [kidney].

Decliners more often described themselves as risk-averse or trying to mitigate potential risks: “It’s a bit risky so I don’t want to get anything that’s risky for me” [kidney]. Moreover, the classification of HCV-viremic organs as “high-risk” amplified concerns regarding HCV-related health effects, and risk of transmission to loved ones—no matter how small—was unsettling for many: “Even though I know that, you know, you guys told me they have medicine out there for [hepatitis C], it’s not so much about me. I have a wife that, you know what I mean, I would not want to jeopardize her life” [kidney].

Some decliners endorsed less trust in science and associated participating in research with concerns about being experimented upon. For example, one patient described how knowing they would be monitored for potential bad outcomes made them apprehensive about the safety of the transplant, saying, “it’s all still new so I don’t want to be the new guinea pig. I want to be the old guinea pig.” [kidney]. However, this hesitation to trust science/research did not always coincide with a lack of trust in healthcare providers, as input from other providers was sometimes the basis for patients refusing to join a trial. For example, when other providers expressed concerns regarding transplant, patients were more likely to cite these discussions as contributing to their risk assessment and decision to decline (“I talked to [my] doctor…we just didn’t think it was going to be a good decision” [kidney]).

Similar to those who enrolled, patients who declined also frequently relied on input from loved ones and friends. For many patients, the opinions of spouses and parents were key to their decision-making: *“*I asked if ‘what do you think about me taking a high-risk kidney?’ And she was like ‘no, no. We just have to wait our turn” [kidney]. Interestingly, multiple participants who declined the trial described the notion of “waiting their turn” for a donor offer instead of skipping/cutting the line by accepting an HCV-viremic organ.

#### Instinctual Approaches

Some decliners made their decision instinctually: one responded with an emphatic “no”; another considered it “zero right from the start” [kidney]. As with enrollees who made their decision instinctually, decliners also employed additional cognitive decision processes, such as the positivist and risk assessment approaches discussed previously, to support their initial gut reaction. For some, instinctual decisions may be informed by previous negative experiences in healthcare. One participant described how the timing of being contacted following being told that their living donor was not an option influenced their decision:

“I had gotten a prospective donor and he’d gone through all the tests… and then [the clinical team] actually called me back to talk about scheduling a date for surgery…right after that happened, they called and said there was a problem with the kidney, and they could not proceed. And I was kind of like really blown away by that because literally I was weeks away from surgery... And that was right like the next day, a physician called me from [hospital] to see if I wanted to participate in a study for hep C kidney. I was just like, [laughs] I was in no mood to even entertain the possibility, I almost felt like it was a setup. You know, I know it wasn’t, but here he calls me within 24 hours of me getting one dropped. And so I feel like outright, I was in no mood for that so it was bad timing on his part but that was the frustrating thing for me was getting so close.” [kidney; declined]

## DISCUSSION

We conducted an exploratory qualitative study among HCV-negative kidney or lung transplant candidates who were approached for enrollment in a clinical trial evaluating the safety and efficacy of HCV-viremic organ transplant. Three decision-making approaches emerged: positivist, risk analyses, and instinctual response. These approaches were mapped to the “attitudes,” “norms,” and “perceived behavioral control” domains of the IBM. Multiple decision-making approaches were associated with each IBM domain, suggesting that decision-making approaches can complement each other. Patients relied upon various sources of information, including research and statistics, advice from healthcare professionals and social networks, and their own emotions and decisional autonomy. Similar decision-making approaches were found among enrollers and decliners; however, outcomes differed based on the meaning that participants assigned to the information that they had available. Moreover, most patients relied on more than one approach, either simultaneously or sequentially, such as supporting an instinctual decision with additional data.

Only one other study^26^ has explored the experiences of HCV-negative patients who received transplant with HCV-viremic organs. Van Pilsum Rasmussen et al^26^ interviewed 8 posttransplant patients and reported that participants weighed risks/benefits of transplant with HCV-viremic organs in their decision. Our study builds on these findings by interviewing trial decliners in addition to enrollees, as well as pretransplant and posttransplant patients. We similarly found that patients considered multiple factors, including shorter wait-time, impact of dialysis on health and quality of life, donor age, graft viability, and HCV-related health complications. Participants described various concerns regarding HCV risk, including potential for developing chronic HCV infection and likelihood of cure, the possibility of associated coinfections (eg, HIV), medication side-effects, long-term health consequences (eg, liver disease and complications of existing comorbidities), and social consequences (eg, transmission to others, disease stigma). Trust in science also influenced how patients framed available data and their perception of contributing to research versus being experimented upon. Participants frequently expressed concerns within the context of their overall illness-experience, such as their conception of their pretransplant health, understanding of the transplant process and inherent risks, and posttransplant goals and prognostication.

Participants who employed *positivist* approaches referenced clinical trial data and statistics when evaluating potential outcomes, including the impact of HCV on transplant success (eg, organ function, rejection risk) and long-term health prognosis. Both participants who enrolled and those who declined discussed the robustness of current data (eg, sample size and follow-up duration); they also desired population outcome statistics. However, patients differed in the meaning they assigned to this information and faced challenges when trying to apply these data to their own health circumstances to gauge how transplantation with HCV-viremic donor organs might impact their prognoses. Thus, in addition to presenting current data to patients, it is critical that education sessions help patients interpret and frame this data within their own health context.^48^

Patients who employed *risk assessment* similarly used outcome data in their decision-making, but their decisions relied on more complex and nuanced calculations of multiple potential benefits and risks. Many patients conceptualized risks associated with HCV-viremic organs as additive to preexisting transplant risk, whereas others considered tradeoffs in risk between options; many further defined personal thresholds for acceptable risk. Although most patients acknowledged inherent risks of the transplant procedure and the potential for posttransplant complications, the way patients framed these risks differed between those who joined or declined the trial. Enrollees pointed to risk-tradeoffs and potential benefits, including potentially receiving an organ sooner (due to a larger pool of available organs), having a younger—and possibly healthier—donor, and avoiding health complications associated with remaining on the waitlist. Alternatively, decliners tended to dismiss the benefits of transplant with HCV-viremic organs more quickly while ascribing less risk to longer waitlist and dialysis time. Some transplant risks were also framed differently by lung and kidney patients. For example, lung patients felt that longer wait-time signified the need to increase the size of their donor pool by accepting HCV-viremic donor organs, whereas kidney patients tended to view longer wait-time as indicative of being “closer” to receiving a non-HCV-viremic donor organ. These differences may arise because kidney allocation includes the amount of time spent waiting and/or on dialysis, so that patients who have waited longer often receive higher priority for transplant; conversely, lung allocation (ie, patients’ Lung Allocation Score [LAS]) excludes wait-time (although wait-time can be used in the event patients’ LAS scores are tied).^49^ Such differences also suggest that more education around the impact of wait-time on chances of receiving transplantation and posttransplant outcomes may be necessary to ensure that patients can make an informed decision regarding whether or not to accept HCV-viremic donor organs.

We found that *instinctual* decision-making was prominent among some participants. Though many participants relied more heavily on cognitive decision-making models, some described reaching a decision almost immediately. This is consistent with other research on transplant decisions,^25,48,50,51^ as well as research on trial participation in the nontransplant setting.^52^ Frequently, both enrollees and decliners subsequently employed other cognitive decision models to support their initial decisions. Underlying emotions such as fear and feeling overwhelmed, as well as positive thinking and self-efficacy, modified patients’ perceptions of their own decision-making process and transplant experience.^25,48,51^ Importantly, although instinctual decision-making may parallel an emotional response, most patients who specifically described strong emotions—for example, feeling “overwhelmed,” “scared,” or “leery”—relied more heavily on positivist and risk assessment approaches. For patients who rely on instinctual approaches, it may be especially important to determine (1) whether an emotional response parallels or undergirds their instinctual decision and (2) to the extent that this emotional response is perceived to relate to the clinical trial team, how clinicians might communicate with these patients to ensure adequate sharing and understanding of information.

Recognizing the role of emotions in cognitive decision-making models is important in promoting patient-centered communication during informed consent and shared decision-making.^48,50^ During the informed consent process, information on risks and benefits is communicated with the goal of enabling patients to make rational decisions about their treatment options.^50,53^ Although this approach fulfills the legal definition of informed consent by providing an *objective standard* of the information needed to make a reasonable decision, it may fail to provide the *subjective* information that each particular patient may require to facilitate shared decision-making.^50,54,55^ This is especially true when emotional or instinctual responses are intertwined with risk perceptions, as patients’ subjective thresholds of “acceptable” risk may differ from clinicians’ perspectives on risk (and from the objective standard provided during informed consent).^51^ Currently, informed consent in the context of HCV-viremic donor organ transplant focuses on the presentation and timing of HCV education^32^ and tradeoffs between organ quality, infection risk, wait-time, and posttransplant survival.^56,57^ However, these studies primarily adopt a risk-benefit assessment approach, which, as our study indicates, may not be helpful for all patients. Our study suggests that ignoring alternative decision-making approaches and the context in which they are employed could undermine trust and hinder information sharing between patients and providers. Patient-centered education should therefore be tailored to consider both emotional and cognitive perspectives of patients to promote shared decision-making and patient agency in transplant. Having such discussions within the context of an established patient–provider relationship could strengthen trust among patients and providers and facilitate the informed consent process.

Our study had some limitations. First, this study was conducted within the context of a clinical trial, which implies that our findings may not be generalizable to other settings. However, given that transplantation with HCV-viremic organs into uninfected recipients was generally considered an experimental practice during the time the study was conducted, understanding patient decision-making in the clinical trial context is a reasonable first step in understanding decision-making. Second, as participants were recruited from one clinical trial site, our findings may not capture the concerns, values, and diversity of all transplant candidates. However, the distribution of demographic characteristics of respondents in our study reflects that of the broader clinical trials in which they were conducted^10,18,19^ and of other qualitative studies of HCV-viremic organ recipients.^26^ Additionally, we aimed to maximize representativeness by including more than one transplant population (kidney and lung) and by interviewing patients at different transplant stages (pretransplant, posttransplant, and declined). Third, there is a potential for recall bias, especially among posttransplant respondents, who were interviewed after their recovery from transplant surgery. We sought to overcome this concern by probing respondents to elicit complete stories.

Our study also had several strengths. This is the first study to examine patients’ perceptions at multiple stages of transplantation and to evaluate how patients make decisions across decision-outcomes (ie, trial enrollment versus declination), thereby enhancing the robustness of our conceptual model. Our large sample size enabled us to achieve saturation in important subgroups such as pretransplant patients and those who declined trial participation. Finally, including both kidney and lung transplant candidates enabled us to investigate how a specific clinical context may inform decision-making and identify themes that resonate across organs.

Overall, our study illustrates the importance of recognizing the variety of approaches patients employ when making decisions about transplant with HCV-viremic organs, including positivist, risk assessment, and instinctual response. Most patients relied on multiple decision-making approaches either simultaneously or sequentially. Instinctual responses were common, and patients often employed a second approach to support these responses. Given how emotions influence patients’ decision-making, transplant teams should consider tailoring education and informed consent processes to meet patients’ emotional and cognitive perspectives. Such modifications may promote shared decision-making among transplant patients and providers.

## ACKNOWLEDGMENTS

The authors would like to thank Adam Mussell for his help in the administration of this study. We would also like to acknowledge the MYTHIC investigators, especially Meghan Sise and Ray Chung at Massachusetts General Hospital, for their insights into early study design and feasibility.

## Supplementary Material


