# Supplementary material for: Decision-making Among Hepatitis C Virus-negative Transplant Candidates Offered Organs from Donors with HCV Infection
Source: Transplant Direct. 2022 Jul 19;8(8):e1341. doi: 10.1097/TXD.0000000000001341 (PMC9298473; doi:10.1097/TXD.0000000000001341)
Supplement: Supplementary file 1 [file txd-8-e1341-s001.pdf]

SUPPORTING INFORMATION

SDC I: Trial Screening Criteria

Table S1.A: Complete inclusion and exclusion criteria for participants in the THINKER trial (kidney transplant)

Inclusion	Exclusion
Must be waitlisted for a kidney transplant (dialysis is not a requirement if a patient is waitlisted)	Hepatocellular carcinoma
Listed for an isolated kidney transplant with ≤2555 days of accrued transplant waiting time and/or ≤2555 days of dialysis time for blood group A, B, or O, by enrollment	Patients with primary focal segmental glomerulosclerosis (FSGS), FSGS recurring after previous transplant, or disease process with increased risk of causing early graft failure as per the treating nephrologist
Listed for an isolated kidney transplant with ≤1825 days of accrued transplant waiting time and/or ≤1825 days of dialysis time for blood group AB, by enrollment	HIV positive
No available living kidney donor	HCV RNA positive (can be isolated HCV antibody positive provided the participant has no history of previously treated HCV)
Between 30-70 years of age, by enrollment	Hepatitis B surface antigen positive
Have a panel reactive antibody level ≤97%	Any other chronic liver disease (excluding non-alcoholic fatty liver disease (NAFLD) with abnormal liver enzymes
eGFR <15ml/min/1.73m2 as calculated using the 4 variable MDRD equation	Persistently elevated liver transaminases
Obtained agreement for participation from the patient's treating transplant nephrologist	Significant hepatic fibrosis on screening elastography (≥f2 fibrosis)
Able to travel to the University of Pennsylvania for routine post-transplant visits and study visits for a minimum of 6 months after transplantation	Pregnant or nursing (lactating) women
No active illicit substance abuse	Known allergy or intolerance to tacrolimus that would require post-transplant administration of cyclosporine, rather than tacrolimus given the drug-drug interaction between cyclosporine and Zepatier
Weigh at least 50kg	Waitlisted for a multi-organ transplant (e.g., pancreas-kidney, heart-kidney, etc.)
Women must agree to use birth control in accordance with Mycophenolate Risk Evaluation and Mitigation Strategy (REMS) following transplant due to the increased risk of birth defects and/or miscarriage	Significant cardiomyopathy defined as either:<br>• Left ventricular ejection fraction <40% on most recent echocardiogram<br>• Left ventricular ejection fraction ≥40% but <50% on most recent echocardiogram with an <5 METS of exercise tolerance<br>• Reversible ischemia on stress testing without revascularization
Both men and women must agree to use at least one barrier method to prevent any secretion exchange	
*Inclusion criteria for treatment (not for entry as study patient) will include any detectable HCV RNA	
Able to provide informed consent	

Table S1.B: Complete inclusion and exclusion criteria for participants in the MYTHIC trial (kidney transplant)

Inclusion	Exclusion
21-65 years of age	HCV RNA positive
Able to understand and adhere to the study visit schedule and all other protocol requirements, and must voluntarily sign and date an informed consent	Primary focal segmental glomerulosclerosis (FSGS) or disease process with increased risk of causing early graft failure as assessed by the transplant nephrologist and/or investigator team
No available medically acceptable, compatible living kidney donor	Female who is pregnant, breastfeeding, or is planning to become pregnant during the course of the study
Participant must agree to use an effective method of birth control per protocol specifications	History of severe, life-threatening. or other significant sensitivity to immunosuppressants utilized in kidney transplant
Estimated glomerular filtration rate(eGFR) <15 ml/min/1.73 m^2	Transplant candidate requiring antibody desensitization protocol for transplantation
Listed for an isolated kidney transplantation	
	History of HIV
	Presence of clinically significant liver disease
	HBV surface Ag-positive or detectable HBV DNA
	Most recent calculated panel reactive antibody (cPRA) >80%
	Prior recipient of a non-renal solid organ transplant

Table S1.C: Complete inclusion and exclusion criteria for participants in the SHELTER trial (lung transplant)

Inclusion	Exclusion
18-67 years of age	Hepatocellular carcinoma
Obtained agreement for participation from the lung transplant team	HIV positive
No evident contraindication to lung transplantation other than the underlying lung disorder	HCV RNA positive
Able to travel to the University of Pennsylvania for routine post-transplant visits and study visits for a minimum of 12 months after transplantation	Any chronic liver disease (excluding non-alcoholic fatty liver disease (NAFLD)) that is occurring in the setting of persistently elevated liver enzymes (patients with Alpha-1-antitrypsin lung disease without hepatic involvement are eligible)
No active illicit substance abuse	Hepatitis B surface antigen and/or DNA positive
Women must agree to use birth control in accordance with Mycophenolate Risk Evaluation and Mitigation Strategy (REMS) following transplant due to the increased risk of birth defects and/or miscarriage	Significant fibrosis (≥F2 on the Fibroscan)-for patients with cystic fibrosis, the cutoff will be 11kPa (cutoff for F2 for patients with chronic cholestatic liver disease), whereas for all other patients the cutoff will be 8kPa (the cutoff for fatty liver disease used in the THINKER study).
Both men and women must agree to use at least one barrier method of birth control or remain abstinent following transplant due to risk of HCV transmission	Known allergy or intolerance to tacrolimus that would require post-transplant administration of cyclosporine, rather than tacrolimus given the drug-drug interaction between cyclosporine and Zepatier/Epclusa
Inclusion criteria for treatment (not for entry as study patient) will include any detectable HCV RNA by week 4 post-lung transplantation	Pre-transplant treatment with amiodarone given the drug-drug interaction between amiodarone and Epclusa

	Pregnant or nursing (lactating) women
	Waitlisted for a multi-organ transplant
	Patients with underlying liver disease with or without liver cirrhosis
	Patients with cystic fibrosis who have underlying liver disease
	Re-transplant candidate
	Use of ECMO or mechanical ventilation as a bridge to lung transplantation
	Inability to provide study consent
	Chronic kidney disease with GFR<50 ml/min/1.73 m^2
**Relative contraindications for study participants that will be reviewed on a case-by-case basis by the Lung Transplant Selection Committee and the Principal Investigators**	
Evidence of end organ damage due to diabetes (e.g. retinopathy, nephropathy, ulcerations) and /or brittle diabetes mellitus (e.g. history of diabetic ketoacidosis) and/or uncontrolled diabetes as evidence by a HgbA1C of 7.5-8.5	
Hematologic: Significant coagulation abnormalities, and/or bleeding diatheses	
Active or recent solid or liquid malignancy in the past 5 years (apart from select skin malignancies)	
Patient refusal to receive blood products or transfusions during lung transplant surgery	
Psychosocial: Profound neurocognitive impairment with absence of social support	
Active mental illness or psychosocial instability	
Inadequate insurance and/or financial support for post-transplant care.	
Evidence of drug, tobacco or alcohol abuse within the past six months and failure to satisfy recommended therapy/services/parameters as indicated by social work staff and/or consult team	
History of chronic non-adherence to medical recommendations and/or medications	
PRA >10%	
Severe malnutrition, BMI <18	
Major chronic disabling comorbidity (e.g., lupus, severe arthritis, neurologic diseases, previous stroke with profound residual)	
Symptomatic or severe vascular disease (History of CABG, Aorta-femoral surgery)	

SDC II: Clinical Trial Education Session

All participants in the SHELTER, MYTHIC, and THINKER trials underwent careful education about the risks of hepatitis C virus infection, which was standardized through the use of an IRB approved PowerPoint slide deck and reinforced through the informed consent document. The education covered basic information about the virus and its mode of transmission; risks to the liver; sensible precautions in the household for an infected person (e.g., not sharing razors; using barrier sexual protection); side effects of antiviral medications; data about HCV cure rates in the general population setting; and the potential risks of non-cure. Education was conducted by investigators designated to the IRB, all of whom are practicing transplant clinicians.

Table S2: Characteristics of participants, by clinical trial.

Characteristics	MYTHIC/THINKER (kidney transplant) (n = 33)	SHELTER (lung transplant) (n = 11)
**Time Elapsed (median [IQR])**		
Days between index date* & interview	268 (64, 345)	363 (63, 560)
Days between clinical trial enrollment date** & interview	254.5 (167, 349)	404 (124, 560)
**Age (in years) at time of interview (median [IQR])**	55.0 (51.0, 60.0)	54.0 (48.0, 61.0)
**Sex (n, %)**		
Male	26 (79%)	5 (45%)
Female	7 (21%)	6 (55%)
**Race (n, %)**		
Asian	2 (6%)	0 (0%)
Black / African American	12 (36%)	0 (0%)
White	17 (52%)	10 (91%)
Other / Missing Value	2 (6%)	1 (9%)
**Ethnicity (n, %)**		
Not Hispanic / Latino	31 (94%)	11 (100%)
Hispanic / Latino	2 (14%)	0 (0%)
**Education (n, %)**		
Less than high school degree	1 (3%)	1 (9%)
High school degree / GED	5 (15%)	1 (9%)
Associated degree / Some college	8 (24%)	4 (36%)
College / Graduate degree	14 (42%)	3 (27%)
Other / Missing Value	5 (15%)	2 (18%)
**Employment (n, %)**		
Unemployed or Disability	13 (39%)	9 (82%)
Employed	11 (33%)	2 (18%)
Retired	6 (18%)	0 (0%)
Other / Missing Value	3 (9%)	0 (0%)
**Living Situation (n, %)**		
Alone	3 (9%)	0 (0%)
Lives with spouse or significant other	20 (61%)	8 (73%)
Lives with other family	5 (15%)	3 (27%)
Other / Missing Value	5 (15%)	2 (11%)
**Underlying Etiology of End-Organ Failure**		
COPD	-	5 (45%)
Other lung disease (bronchiectasis, alpha1 antitrypsin, rheumatic, cystic fibrosis)	-	6 (55%)
Diabetes and/or hypertensive nephropathy	16 (48%)	-
IgA nephropathy	7 (21%)	-
Polycystic kidney disease	6 (18%)	-
Other renal (anti-GBM, drug-induced, multifactorial)	4 (12%)	-
**Dialysis Regimen**		-
No dialysis	2 (6%)	-

Peritoneal dialysis	9 (27%)	-
Hemodialysis	22 (67%)	-

Abbreviations: anti-GBM=Anti-Glomerular Basement Membrane; GED=General Educational Development

*Index Date refers to the date each participant was first contacted by the clinical trial team, and is applicable to all study participants.

** Enrollment Date refers to the date each participant completed clinical trial screening or the education session (whichever occurred first), and is only applicable to participants enrolled in the clinical trial.

SDC III: Conceptual Model

Our approach was informed by the Integrated Behavior Model (IBM), a model of health behavior that seeks to understand how patient's attitudes, perception of social norms, and personal agency influence their adoption of a behavior, given sufficient knowledge, salience, and resources in the absence of external or environmental constraints. In this study, we adapted this model (Figure S1) to guide our investigation of: 1) differences in attitudes and beliefs between those who accepted organs from HCV-viremic donors and those who declined, and 2) patient experiences with transplant.

Figure S1. Conceptual model informing qualitative interview domains, adapted from the Integrated Behavior Model (IBM).

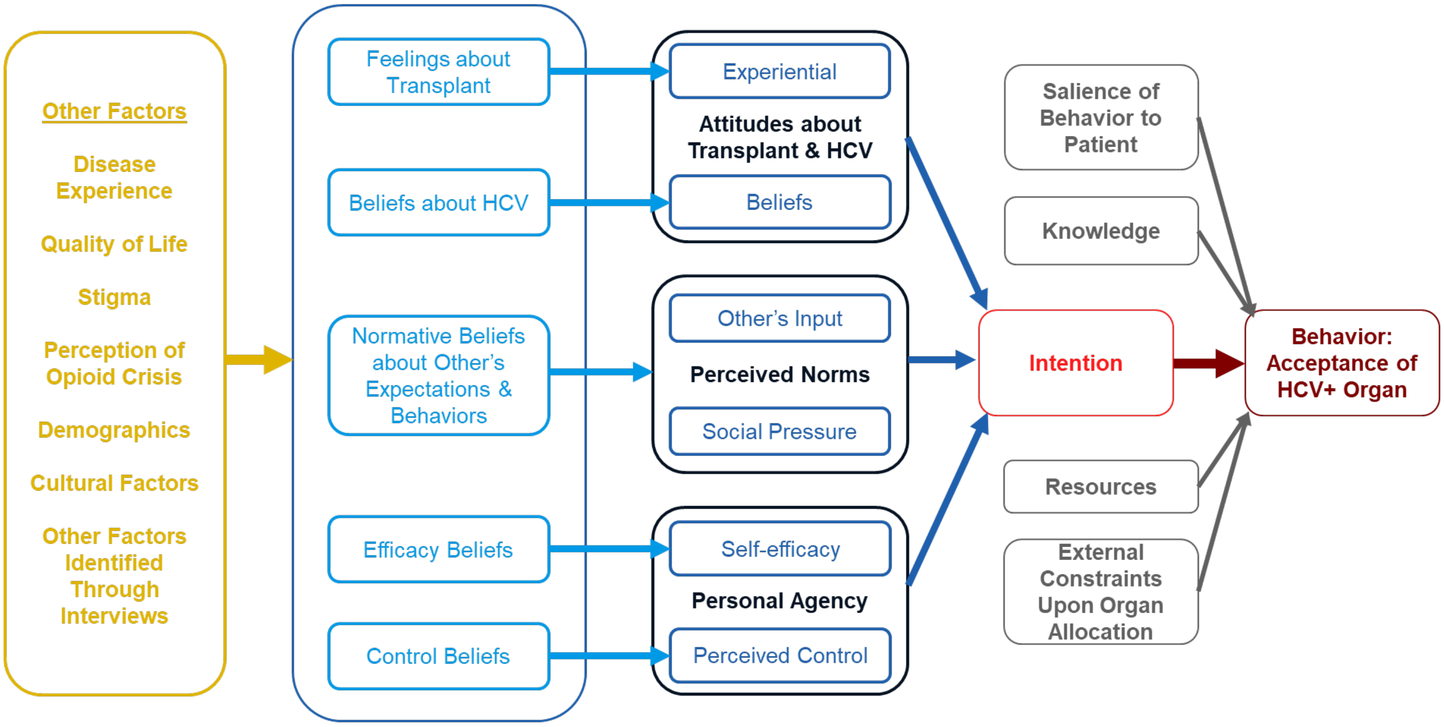

Three decision-making approaches emerged from our analyses: positivist, assessing risks, and instinctual response. These decision-making approaches were employed by most

patients, regardless of whether they enrolled or declined the trial. Contextual factors influencing decisions included attitudes toward research, input from others, waitlist time, and perceptions of organ quality. After incorporating these themes into the IBM model, we obtain the following conceptual model (Main Text Figure 2). This conceptual model is stratified by patients' choice to enroll in or decline the trial (or, equivalently, by patients' intention to receive organs from HCV-viremic donors or not, respectively). In each stratum, multiple decision-making approaches were associated with each IBM domain. For example, positivist and risk assessment approaches reflected patients' attitudes and perceptions of social norms, regardless of whether patients ultimately chose to enroll or decline the trial. Instinctual responses frequently exemplified perceived behavioral control, although they sometimes also reflected patients' attitudes and perceived social norms, complementing positivist and risk assessment approaches to decision-making. These observations suggest that decision-making approaches are not mutually exclusive and can be employed by all patients, regardless of whether they intend to receive organs from HCV-viremic donors or not.

SDC IV: Interview Guide

Participants were asked all questions unless otherwise indicated.

Domain 1: Experience with Underlying Illness

Q1) Thinking about the first time you met with the transplant program, could you walk me through what that was like?

Probes: What sorts of thoughts were in your mind at the time?
How did you feel when your doctor discussed that you would need a [lung/kidney] transplant?

Q2) Think about the aspects of your life that really matter to you. What are these things? How did needing a [lung / kidney] transplant impact them?

Kidney transplant patients (MYTHIC/THINKER):
2a) How did dialysis affect these things?

Domain 2: Decision Making and Perceptions of Hepatitis C Virus

Q3) Think back to when you made the decision to enroll in the SHELTER/MYTHIC/THINKER trial. What did you think about getting a [lung / kidney] from someone with hepatitis C?

Probes: Did you have specific concerns about receiving a hepatitis C infected [lung/kidney]?

Q4) Can you walk me through how you came to your decision regarding transplant with a [lung / kidney] from hepatitis C-positive donor?

Probes: Was there anything specifically that helped you make up your mind?

Q5) Were there people in your life who you turned to for advice on whether you should consider a [lung / kidney] with hepatitis C?

Probes: What did you talk about in these conversations?
How much do you think their input influenced your decisions?
Are there specific things they said that stuck with you?
Have you discussed any of these things with your doctor?

Q6) How did the education session impact your decision to receive a transplant from a donor with hepatitis C?

Probe: Did the education session change how you thought about hepatitis C?
Did anything you were told surprise you?
Was there anything specifically that helped you make up your mind?

Domain 3: Transplant Experience *(post-transplant participants only)*

Q7) Think about the day you were called to come in for your [lung/kidney] transplant. Can you walk me through what happened that day?

Probes: What do you remember thinking about after you woke up from surgery?

Q8) What did the doctors tell you about whether or not you needed to be treated for hepatitis C after the transplant?

Probes: How did you feel when you heard this?

Q9) We are interested in how patients think about hepatitis C as part of their overall health following transplant with a [lung / kidney] from a hepatitis C positive donor. If you were going to talk to a new doctor about your medical history, would you tell them about hepatitis C?

Probes: What would you tell them?
Do you have any concerns about hepatitis C and your health for the future?

Q10) How do you think that your feelings about hepatitis C have changed since your transplant?

Q11) Let's return to thinking about the aspects of your life that really matter to you. So far, how has your [lung / kidney] transplant impacted these things?

Probes: Are there things you can do now that you couldn't do before transplant?
What changes have surprised you?

Domain 4: Experience in Clinical Trial

Q21) How has your experience in the [SHELTER/MYTHIC/THINKER] trial been?

Probes: Do you think being part of the trial has impacted how you feel about your transplant experience?

Q13) Imagine your doctors are talking to another patient who might be interested in receiving a transplant from a donor with hepatitis C. What sorts of information should be communicated to the patient to help them decide if they want to accept or decline transplant from a donor with hepatitis C?

SDC V: COREQ Checklist

This study adhered to the recommendations provided in the Consolidated Criteria for Reporting Qualitative Studies [COREQ] checklist. The completed checklist appears below.

COREQ (COnsolidated criteria for REporting Qualitative research) Checklist

A checklist of items that should be included in reports of qualitative research. You must report the page number in your manuscript where you consider each of the items listed in this checklist. If you have not included this information, either revise your manuscript accordingly before submitting or note N/A.

Topic	Item No.	Guide Questions/Description	Reported on Page No.
**Domain 1: Research team and reflexivity**			
Personal characteristics			
Interviewer/facilitator	1	Which author/s conducted the interview or focus group?	8
Credentials	2	What were the researcher's credentials? E.g. PhD, MD	8
Occupation	3	What was their occupation at the time of the study?	8
Gender	4	Was the researcher male or female?	8
Experience and training	5	What experience or training did the researcher have?	8
Relationship with participants			
Relationship established	6	Was a relationship established prior to study commencement?	8
Participant knowledge of the interviewer	7	What did the participants know about the researcher? e.g. personal goals, reasons for doing the research	8
Interviewer characteristics	8	What characteristics were reported about the inter viewer/facilitator? e.g. Bias, assumptions, reasons and interests in the research topic	8
**Domain 2: Study design**			
Theoretical framework			
Methodological orientation and Theory	9	What methodological orientation was stated to underpin the study? e.g. grounded theory, discourse analysis, ethnography, phenomenology, content analysis	10
Participant selection			
Sampling	10	How were participants selected? e.g. purposive, convenience, consecutive, snowball	9
Method of approach	11	How were participants approached? e.g. face-to-face, telephone, mail, email	8
Sample size	12	How many participants were in the study?	11
Non-participation	13	How many people refused to participate or dropped out? Reasons?	11
Setting			
Setting of data collection	14	Where was the data collected? e.g. home, clinic, workplace	8
Presence of non-participants	15	Was anyone else present besides the participants and researchers?	8
Description of sample	16	What are the important characteristics of the sample? e.g. demographic data, date	8, 11-12
Data collection			
Interview guide	17	Were questions, prompts, guides provided by the authors? Was it pilot tested?	9-10
Repeat interviews	18	Were repeat inter views carried out? If yes, how many?	N/A
Audio/visual recording	19	Did the research use audio or visual recording to collect the data?	9-10
Field notes	20	Were field notes made during and/or after the inter view or focus group?	10
Duration	21	What was the duration of the inter views or focus group?	11
Data saturation	22	Was data saturation discussed?	9
Transcripts returned	23	Were transcripts returned to participants for comment and/or	N/A

Topic	Item No.	Guide Questions/Description	Reported on Page No.
		correction?	
**Domain 3: analysis and findings**			
Data analysis			
Number of data coders	24	How many data coders coded the data?	10
Description of the coding tree	25	Did authors provide a description of the coding tree?	10
Derivation of themes	26	Were themes identified in advance or derived from the data?	10
Software	27	What software, if applicable, was used to manage the data?	10
Participant checking	28	Did participants provide feedback on the findings?	N/A
Reporting			
Quotations presented	29	Were participant quotations presented to illustrate the themes/findings? Was each quotation identified? e.g. participant number	TABLE 3
Data and findings consistent	30	Was there consistency between the data presented and the findings?	12-15
Clarity of major themes	31	Were major themes clearly presented in the findings?	12-15
Clarity of minor themes	32	Is there a description of diverse cases or discussion of minor themes?	12-15

Developed from: Tong A, Sainsbury P, Craig J. Consolidated criteria for reporting qualitative research (COREQ): a 32-item checklist for interviews and focus groups. *International Journal for Quality in Health Care*. 2007. Volume 19, Number 6: pp. 349 – 357

Once you have completed this checklist, please save a copy and upload it as part of your submission. DO NOT include this checklist as part of the main manuscript document. It must be uploaded as a separate file.
